# Is It Possible to Establish Thresholds for Depressive Symptom Severity Associated With Cognitive Impairments in Bipolar Disorders? Findings From the FACE‐BD Cohort

**DOI:** 10.1155/da/9918396

**Published:** 2026-10-26

**Authors:** Nathan Vidal, Eric Brunet-Gouet, Alexandre Billard, Solène Frileux, Raoul Belzeaux, Anamaria Bogdan, Philippe Courtet, Thierry D’Amato, Caroline Dubertret, Bruno Etain, Emmanuel Haffen, Dominique Januel, Marion Leboyer, Antoine Lefrere, Pierre-Michel Llorca, Emeline Marlinge, Katia M’Bailara, Emilie Olié, Mircea Polosan, Raymund Schwan, Michel Walter, The FACE-BD (FondaMental Academic Centers of Expertise for Bipolar Disorders) group, Christine Passerieux, Paul Roux

**Affiliations:** ^1^ Fondation FondaMental Créteil France; ^2^ DisAP MOODS Team INSERM UMR1018 CESP Université de Versailles Saint-Quentin-En-Yvelines Université Paris-Saclay Le Chesnay France universite-paris-saclay.fr; ^3^ Centre Hospitalier de Versailles Service Universitaire de Psychiatrie d’Adultes et d’Addictologie Le Chesnay France; ^4^ CHU Montpellier France chru-strasbourg.fr; ^5^ IGF University of Montpellier CNRS INSERM Montpellier France inserm.fr; ^6^ Pôle de Psychiatrie Centre Hospitalier Princesse Grace Monaco Monaco chpg.mc; ^7^ Department of Emergency Psychiatry and Acute Care Lapeyronie Hospital CHU Montpellier Montpellier France chu-montpellier.fr; ^8^ INSERM U1028 CNRS UMR5292 Lyon Neuroscience Research Center Psychiatric Disorders: From Resistance to Response Team University Lyon 1 Villeurbanne Lyon France univ-lyon1.fr; ^9^ Université de Paris Inserm UMR1266 Sorbonne Paris Cité Faculté de Médecine Paris France sorbonne-paris-cite.fr; ^10^ AP-HP Groupe Hospitalo-Universitaire AP-HP Nord DMU ESPRIT Service de Psychiatrie et Addictologie Hôpital Louis Mourier Colombes France aphp.fr; ^11^ Optimisation Thérapeutique en Neuropharmacologie OPTEN U1144 INSERM Université Paris Cité Paris 75006 France u-paris.fr; ^12^ Département de Psychiatrie et de Médecine Addictologique Hôpitaux Lariboisière-Fernand Widal GHU APHP Nord Université Paris Cité Paris F-75010 France u-paris.fr; ^13^ UMR INSERM 1322 LINC Université Marie et Louis Pasteur Besançon France; ^14^ Service de Psychiatrie de l’adulte CIC-1431 INSERM CHU de Besançon Besançon F-25000 France; ^15^ Université Sorbonne Paris Nord Bobigny Cedex 93017 France univ-paris13.fr; ^16^ Département de Recherche Clinique Pôle Universitaire 03 G03 EPS Ville Evrard Neuilly sur Marne France; ^17^ Inserm U 955 Neuropsychiatrie Translationnelle DMU IMPACT (AP-HP) Département de Psychiatrie, Fondation FondaMental Université Paris Est Créteil (UPEC) Créteil 94000 France u-pec.fr; ^18^ Pôle de Psychiatrie Assistance Publique Hôpitaux de Marseille Marseille France ap-hm.fr; ^19^ NT-UMR7289 CNRS Aix-Marseille Université Marseille France; ^20^ Centre Hospitalier et Universitaire Département de Psychiatrie EA 7280 Université d’Auvergne Clermont-Ferrand France u-clermont1.fr; ^21^ Centre Hospitalier Charles Perrens Pôle de Psychiatrie Générale et Universitaire Bordeaux France; ^22^ LabPsy UR 4139 Université de Bordeaux Bordeaux F-33000 France u-bordeaux.fr; ^23^ Université Grenoble Alpes Inserm U1216 CHU Grenoble Alpes Grenoble Institut Neurosciences Grenoble France univ-grenoble-alpes.fr; ^24^ Centre Psychothérapique de Nancy Inserm U1254 Université de Lorraine Nancy France univ-lorraine.fr; ^25^ Service Hospitalo-Universitaire de Psychiatrie Générale et de Réhabilitation Psycho Sociale 29G01 et 29G02 CHRU de Brest Hôpital de Bohars Brest France; ^26^ Service de Psychiatrie du Secteur 78G18 Centre Hospitalier de Plaisir Plaisir France

**Keywords:** bipolar depression, bipolar disorders, cognition, depression

## Abstract

Individuals with bipolar disorders (BDs) with depressive symptoms exhibit poorer cognitive performance and are often excluded from procognitive interventions and cognitive research. Threshold values on depression severity scales, based on cutoffs defining depressive episodes, have traditionally been used to exclude patients suspected of having depression‐associated cognitive impairment. Identifying a threshold value for depression‐associated cognitive impairment on these scales could help guide patient selection and treatment. We assessed neuropsychological performance in outpatients with BD with varying levels of depressive symptom severity measured by the Montgomery–Åsberg Depression Rating Scale (MADRS). The sample was divided based on the total MADRS score or subscores in MADRS theoretical domains. We then compared average cognitive performance between groups while adjusting for relevant covariates to determine a MADRS score above which cognition is significantly impaired. We included 2532 individuals with BD. The composite cognitive score was significantly lower in individuals with a MADRS score ≥ 12 than in those with a score ≤ 1 but did not differ from that of individuals with scores between 2 and 11. Symptoms of detachment and sadness were more strongly associated with cognitive impairment than other symptoms. Individuals with type I BD with MADRS detachment subscores higher than 4 and individuals with type II BD with nonzero sadness subscores had significant cognitive impairment compared to individuals with lower subscores. Excluding individuals with BD whose cognition may be affected by depressive symptoms would require omitting those with MADRS detachment subscores higher than 4 or nonzero sadness subscores. Our findings question the existence of a total depression severity threshold for cognitive impairment, as specific depressive symptoms seem more strongly associated with cognitive impairment than total symptom severity.

## 1. Introduction

Individuals with bipolar disorders (BDs) can exhibit cognitive impairment, especially in verbal memory and executive function, that can persist between mood episodes [1–3]. Poorer cognitive performance is significantly associated with worse psychosocial functioning [3, 4], more hospitalizations, manic or hypomanic episodes [5], and suicide attempts in BD [6]. Individuals with bipolar depression exhibit poorer verbal fluency [6, 7], verbal learning [8], psychomotor speed [9], attention [6, 8, 10], and working memory [10] than euthymic patients. Outside of a depressive episode, mild or moderate residual depressive symptoms may still contribute to cognitive impairment [8], particularly in verbal fluency [11], psychomotor speed, processing speed, attention [12], executive function, and memory [13]. Identifying a depressive symptom severity level at which cognitive impairment emerges could provide valuable insights for clinical and research settings. Indeed, such a threshold may guide the inclusion of individuals into procognitive interventions that do not target the improvement of residual bipolar depression.

The gold standard for diagnosing a major depressive episode is through a structured clinical interview for the DSM‐IV‐R (SCID) conducted by a trained clinician, who determines whether the DSM criteria are met [14]. However, a key limitation of the DSM criteria is the lack of quantitative measures of the recovery from a mood episode (American Psychiatric [15]). Various rating scales, such as the Montgomery–Åsberg Depression Rating Scale (MADRS) [16], have been commonly used in BD to provide a continuous measure of depressive symptom severity and to distinguish euthymia from depression. The International Society for BDs proposed that a MADRS score ≤ 5 or 7 indicates remission, a score between 8 and 14 indicates subsyndromal depression, and a score > 14 indicates syndromal depression [17]. These thresholds were determined by the strength of their association with the global severity of depression, measured by the clinical global impression scale (CGI‐S) [18] or the standardized clinical outcome ratings (SCOR), that is, the number of DSM criteria met [19]. As a result, MADRS scores align very well with DSM’s definition of depression: A MADRS score greater than 14 effectively discriminates nondepressed from depressed individuals as diagnosed by the SCID using DSM‐IV criteria with an AUC of 0.90 [20].

When investigating cognitive impairments in BD or conducting procognitive clinical trials, patients scoring above certain thresholds are often excluded to mitigate the cognitive effects of residual depressive symptoms and isolate other factors influencing cognitive performance. For example, Sparding and collaborators excluded participants with MADRS scores of 14 or higher to study cognitive performance only in euthymic patients with BD [21]. In research on cognition, thresholds for depression scales are usually less conservative than those used to define remission to minimize exclusions [22]. However, there is no consensual threshold for depression severity above which depression symptoms affect cognition.

We aimed to identify a threshold value on a depression severity scale that would suggest the development of depression‐induced cognitive impairment in individuals with BD. This threshold could guide the patient orientation in routine care, distinguishing when to prioritize interventions targeting subthreshold depression and when to focus on treatments addressing cognitive impairments, such as procognitive medication or cognitive remediation. Our goal was also to account for confounding factors that may affect cognition, maximizing the specificity of the threshold across individuals with BD.

## 2. Methods

Our cross‐sectional study adhered to the STROBE guidelines for observational study reporting proposed by the EQUATOR team [23] (see SM1). A preprint of the study is accessible online [24].

### 2.1. Participants

We included outpatients from the FACE‐BD (FondaMental Advanced Centers of Expertise for BDs) cohort, recruited between February 2009 and February 2022 by a French national network of 12 centers (Bordeaux, Créteil, Montpellier, Grenoble, Nancy, Marseille, Paris, Versailles, Monaco, Clermont‐Ferrand, Colombes, and Besançon). This network was established by the Fondation FondaMental (https://www.fondation-fondamental.org).

The diagnosis of BD was determined using the structured clinical interview for the DSM‐IV‐R (SCID) criteria [14]. We included outpatients aged 18 and older with type I, type II, or not otherwise specified (NOS) BD. As we focused on the depression severity spectrum, from euthymia to severe depression, we excluded people experiencing a characterized manic, hypomanic, or mixed episode as defined by the DSM‐IV‐R criteria at the moment of testing. We also excluded people with neurological comorbidities (multiple sclerosis, epilepsy, stroke, and other neurological conditions), learning disabilities, substance abuse in the past month, and those who had undergone electroconvulsive therapy within the past year because those factors could have impacted cognition, creating a bias in the results.

### 2.2. Measurements

#### 2.2.1. Mood Symptoms

Among participants, people experiencing a major depressive episode at the moment of testing were identified using the DSM criteria. Additionally, we assessed the total severity of depressive symptoms in all participants using a continuous measurement to evaluate the validity of potential thresholds. We used the MADRS [16]. The MADRS consists of 10 items assessing various aspects of depression. Each item is scored on a 0–6 scale, with higher scores indicating greater symptom severity. The total MADRS score is discrete, varying between 0 and 60. In our sample, the scale showed high internal consistency (McDonald’s *ω* = 0.83). Manic symptoms were assessed by the Young Mania Rating Scale (YMRS) [25]. Trained clinicians from the FACE‐BD network administered both the MADRS and YMRS questionnaires. These ratings were based on the patients’ subjective reports of their clinical state over the past 48 h, along with the clinicians’ observations made during the interview.

#### 2.2.2. Cognition

Neuropsychological tests were administered by experienced neuropsychologists in a standardized order across all centers. Each testing session lasted ~120 min, including 5 to 10 min breaks. The test battery consisted of 11 assessments and complied with standardized guidelines. It included five subtests from the Wechsler Adult Intelligence Scale, either version III (WAIS‐III) [26] or version IV (WAIS‐IV) [27], depending on the availability of the French version of WAIS‐IV over the study period. Our cognitive battery tested five of the six cognitive domains proposed by the International Society for BDs [28] (speed of processing, attention, working memory, verbal learning, and executive functioning), using seven of the 12 cognitive tests (the Trail Making Test, semantic fluency test, the Continuous Performance Test, WAIS span test, the CVLT, and the Stroop test). We investigated a 6th domain in addition to those of ISBD‐BANC, named verbal comprehension and perceptual reasoning, as a proxy for generalized cognitive ability (intellectual functioning/estimated IQ). Our battery is defined as follows.

• Verbal comprehension and perceptual reasoning: assessed with WAIS Vocabulary and Matrices.
• Attention: evaluated through Conners’ Continuous Performance Test II V.5, analyzing omission, commission, variability, and detectability scores [29].
• Processing speed: assessed using digit symbol coding (WAIS‐III) or coding (WAIS‐IV), WAIS symbol search, and the Trail Making Test, Part A [30].
• Working memory: assessed with WAIS digit span (total score) and spatial span (forward and backward scores) from the Wechsler Memory Scale, version III [31].
• Verbal memory: measured by the California Verbal Learning Test, including immediate recall and short and long delay‐free recall [32].
• Executive function: measured using the Stroop test (color/word condition) [33], semantic and phonemic verbal fluency [34], and Trail Making Test, Part B [30].

Higher scores indicated better cognitive performance. Raw scores for each test were converted to standardized scores based on normative data [29, 33, 35, 36]. We computed a mean *z*‐score for each cognitive domain and a composite cognitive score by averaging the scores across cognitive domains except verbal comprehension and perceptual reasoning, which are not included in the tests recommended by the ISBD‐BANC and are not affected by BD [37].

#### 2.2.3. Other Clinical Variables

We recorded patients’ sex at birth (self‐reported), age, BD subtype, and education level. We collected the total number of lifetime mood episodes, the age at BD onset, the duration of the disorder, and the history of psychotic symptoms. Besides, we recorded the use of antidepressants, anticonvulsants, lithium, antipsychotics, anxiolytics, and antiparkinsonians prescribed to treat extrapyramidal side effects. Chlorpromazine equivalents (CPZeqs) were calculated using a formula proposed by Andreasen, Leucht, and collaborators [38, 39]. The anticholinergic burden was assessed using the maximum score of the scale developed by Chew and collaborators [40].

### 2.3. Statistical Analyses

Statistical analyses were performed using *R* version 4.3.1. We hypothesized that the data were missing at random (MAR) and therefore estimated data using multivariate imputation by chained equations (50 imputations, *mice* package of R) [41]. Multiple linear regression analyses were conducted on the imputed datasets, except for models predicting attention performance, which had 33.8% of missing data. Those models were estimated based on complete‐case data. The fraction of missing information (fmi), that is, the proportion of the parameter’s variance attributable to missing data, was reported in the results.

We first estimated linear regression models to predict cognitive performance, with depression defined by DSM criteria as the main predictor. We adjusted those models for education level; the history of psychotic symptoms; anticholinergic burden; the use of anxiolytics, anticonvulsants, antipsychotics, or lithium; the use of any psychotropic treatment; time since the onset of the disorder; and a variable indicating that the patient experienced more than one depressive episode, which can confound the association between depressive symptoms and cognition [42, 43]. Age and sex were accounted for in the standardization of cognitive performance scores.

To assess the shape of the association between the MADRS total score and cognition, we estimated a linear regression model of composite cognitive score using the continuous MADRS total score as a predictor in individuals with and without DSM criteria for major depressive episode. Then, we wanted to identify a cutoff for the MADRS total score such that individuals with scores below the cutoff demonstrated significantly better cognitive performance compared to those with scores above the cutoff. We split patients into six categories based on five cutoff values for the MADRS (≤1, 2–4, 5–7, 8–11, 12–17, and ≥18) using the *R* function *cut_number* so that each category included a similar number of patients deemed sufficient by the power analysis. The power analysis reported that 283 individuals are sufficient to detect an effect size corresponding to *R* ^2^ = 0.07 [12] in a linear regression model including 18 predictors with 80% statistical power. We assessed the effect of belonging to each MADRS score category on composite and domain‐specific cognitive performance using multiple linear regression models, adjusting for the same set of covariates. To identify the MADRS categories characterized by significant cognitive impairment, we reported the contrasts between the effects of each MADRS score category on cognitive performance after controlling for multiple testing by applying Benjamini–Hochberg’s false discovery rate to the *p*‐values, with a significance threshold set at 0.05 [44].

We wanted to assess the existence of cutoffs for MADRS theoretical domain subscores. Therefore, we conducted similar analyses with four theoretical domains of the MADRS: sadness (assessed by apparent and reported sadness), negative thoughts (pessimistic and suicidal thoughts), detachment (concentration difficulties, lassitude, and inability to feel), and neurovegetative symptoms (inner tension, reduced sleep, and reduced appetite) [45]. We summed the scores of each item included in the theoretical domain to obtain a subscore for the domain. Subgroup analyses were conducted across BD subtypes. Finally, to determine whether specific depressive symptoms could explain depression‐associated cognitive impairment, we assessed the correlations between individual MADRS items and cognitive performance by measuring Spearman’s *ρ* coefficients after controlling for multiple testing using the Benjamini–Hochberg’s procedure.

## 3. Results

### 3.1. Description of the Sample

The final sample included 2532 people (62.1% females, aged 39.2 ± 12 years) (flowchart is available in Figure S1) with a mean MADRS total score of 9.3 ± 8.3 (range: 0–45) and a mean YMRS total score of 2.1 ± 3.3 (range: 0–23) (Table 1). Our sample included 47.9% of people with type I BD and 41.6% with type II BD. People in our sample had, on average, an education level of 14.4 years and slightly lower cognitive performance than the norm (mean *z*‐score of −0.24), except for verbal comprehension and perceptual reasoning (Table 2).

**Table 1:** Description of the sample (*n* = 2532).

Category	*n*	Mean (SD)	*n* (%) missing data
Female	1573 (62.1%)	–	2 (<1%)
Age	–	39.2 (12)	0
Education level, years	–	14.4 (2.6)	0
MADRS	–	9.3 (8.3)	0
YMRS	–	2.1 (3.3)	4 (<1%)
Depressive episode	210 (8.3%)	–	0
Type I BD	1212 (47.9%)	–	0
Type II BD	1054 (41.6%)	–	0
Unspecified BD	266 (10.5%)	–	0
End of the last characterized mood episode > 3 months before	1928 (77.2%)	–	35 (1.4%)
Age at the first mood episode	–	23.7 (9.0)	180 (7.1%)
Duration of the bipolar disorder, years	–	15.5 (10.7)	180 (7.1%)
Number of depressive episodes	–	5.7 (5.9)	471 (18.6%)
Number of manic episodes	–	1.2 (2.1)	301 (11.9%)
Number of hypomanic episodes	–	3.4 (5.6)	700 (27.6%)
Patients with a history of psychosis	846 (38.6%)	–	338 (13.3%)
Number of medications	–	2 (1.4)	448 (17.7%)
Number of patients using antidepressants	685 (32.9%)	–	448 (17.7%)
Anticonvulsants	920 (44.2%)	–	448 (17.7%)
Lithium	622 (29.9%)	–	448 (17.7%)
Antipsychotics	815 (39.1%)	–	448 (17.7%)
Anxiolytics	505 (24.3%)	–	448 (17.7%)
Anticholinergic agents	29 (1.4%)	–	448 (17.7%)
Anticholinergic burden, /24 h	–	0.66 (0.69)	448 (17.7%)
Chlorpromazine equivalents mg/24 h	–	95.6 (213.3)	541 (21.4%)

Abbreviations: CGI‐S, clinical global impression severity scale; MADRS, Montgomery–Asberg Depression Rating Scale; YMRS, Young Mania Rating Scale.

**Table 2:** Cognitive performance in the six cognitive domains and over all domains (*n* = 2532).

Category	Mean	SD	*n* (%) missing data
Attention	−0.61	1.39	856 (33.8%)
Executive function	−0.25	1.09	46 (1.8%)
Processing speed	−0.06	0.74	9 (<1%)
Verbal memory	−0.28	1.33	73 (2.9%)
Working memory	−0.10	0.93	41 (1.6%)
Composite score	−0.24	0.73	0
Verbal comprehension and perceptual reasoning	0.51	1.05	590 (23.3%)

*Note*: Values represent demographically corrected *z*‐scores based on normative data.

### 3.2. Cognitive Performance During a Depressive Episode as Defined by the SCID Using DSM Criteria

People experiencing a depressive episode according to the DSM criteria had significantly worse performance in processing speed (standardized *β* = −0.18, adjusted *p* = 0.019), executive function (standardized *β* = −0.22, adjusted *p* = 0.006), working memory (standardized *β* = −0.21, adjusted *p* = 0.007), and composite cognitive score (standardized *β* = −0.21, adjusted *p* = 0.007) (Table 3). Those significant associations persisted only among patients with type I BD (Table S2).

**Table 3:** Association between major depression diagnosis based on the DSM criteria and cognitive performance.

Category	*n*		*β* (95% CI)	Standardized *β*	Adjusted *p*‐value	fmi
	Non‐depressed	Depressed				
Attention	1371	105	−0.19 (−0.54: 0.16)	−0.14	0.284	–
Executive function	2322	210	**−0.25 (−0.42: −0.08)**	**−0.23**	**0.005**	**0.009**
Processing speed	2322	210	**−0.14 (−0.26: −0.03)**	**−0.19**	**0.013**	**0.011**
Verbal memory	2322	210	−0.13 (−0.34: 0.07)	−0.10	0.203	0.024
Working memory	2322	210	**−0.22 (−0.36: −0.07)**	**−0.23**	**0.003**	**0.014**
Composite score	2325	210	**−0.17 (−0.28: −0.06)**	**−0.23**	**0.003**	**0.002**
Verbal comprehension and perceptual reasoning	2322	210	0.07 (−0.17: 0.25)	0.07	0.406	0.251

*Note:* Estimates were obtained in multiple linear regression models. Results for the covariates are displayed in Table S1 for clarity. Bold values indicate the *p*‐Values.

### 3.3. Cognitive Performance and Depression Severity in Individuals With and Without a DSM‐Based Diagnosis of a Major Depressive Episode

We detected a significant but weak linear association between the MADRS total score and the composite cognitive score (*β* = −0.005, *p* = 0.008) (Figure 1).

**Figure 1:**
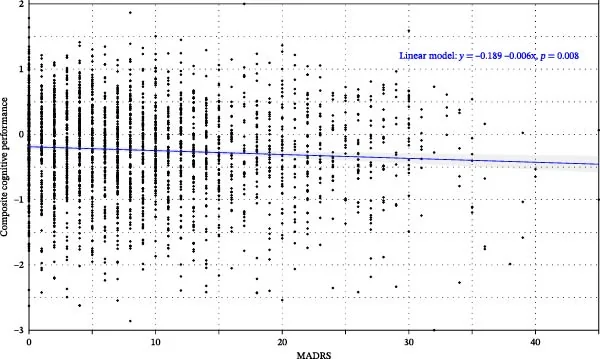
Estimating linear regression model of the association between the MADRS score and global cognitive performance.

To test the existence of a threshold, we built linear regression models of cognitive performance using the MADRS score categories as main predictors while adjusting for the same set of covariates. The results of the model estimating the composite cognitive score are provided in Table S3. We pulled the contrasts between the effects of each MADRS score category on cognitive performance from those models and presented them in Table S4. Patients with a MADRS score ≥ 12 had a significantly lower composite cognitive score than those with a MADRS score ≤ 1, but their performance did not differ significantly from that of patients with a MADRS score between 2 and 11. Similarly, patients with a MADRS score ≥ 5 had significantly lower processing speed performance than patients with a MADRS score ≤ 1, but their performance did not significantly differ from that of patients with a MADRS score between 2 and 4. Notably, 2083 (82.3%) individuals had MADRS scores of 2 or higher. Overall, we were unable to detect clear thresholds for depression‐associated composite cognition or processing speed impairment. In our sample, MADRS ≥ 12 was the best cutoff for classifying depression as defined by the SCID based on DSM criteria (Figure S2). We were unable to detect significant thresholds across BD subtypes (Table S5).

### 3.4. Cognitive Performance and the Severity of Specific Depressive Symptoms

We aimed to assess whether individual depressive symptoms may be associated with cognitive impairment and investigated the existence of cognitive thresholds for domain subscores of the MADRS. The four theoretical domains (sadness, negative thoughts, detachment, and neurovegetative symptoms) fitted well our MADRS data, as demonstrated by a comparative fit index (CFI) above 0.95 (CFI = 0.979) and a root mean square error of approximation (RMSEA) not statistically greater than 0.05 (RMSEA (90% CI) = 0.049 (0.043–0.055), one‐sided *p*‐value of the test of the null hypothesis that the RMSEA equals 0.05: 0.58). Individuals with a nonzero sadness subscore (*n* = 1252, 49.4%) had a worse composite cognitive score than individuals with a zero sadness subscore (Table S6). The significance of this threshold persisted only among individuals with type I BD (Table S7). Individuals with a detachment subscore equal to or higher than 4 (*n* = 1057, 41.2%) had a significantly lower composite cognitive score than individuals with a detachment subscore below 4 (Table S6). The significance of this threshold persisted only among individuals with type II BD (Table S7). To note, 1505 (59.4%) individuals had either a positive sadness subscore or a detachment subscore equal to or higher than 4 in our sample.

The depressive symptom most strongly associated with cognition was concentration difficulty, which was significantly associated with all domains (*ρ* = −0.08 to −0.15) (Figure 2). Poorer processing speed was associated with greater apparent sadness, lassitude, inability to feel, and pessimistic thoughts (*ρ* = −0.07 to −0.1). Poorer executive function was associated with increased apparent sadness, inability to feel, and suicidal thoughts (*ρ* = −0.06 to −0.1).

**Figure 2:**
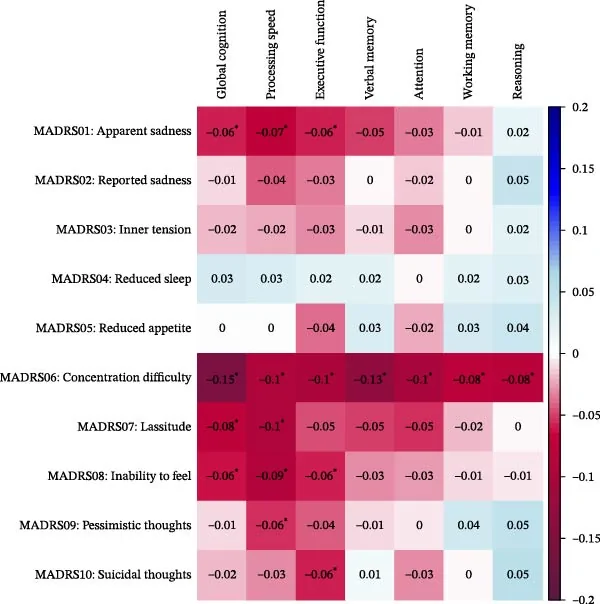
Correlation coefficients between individual items of the MADRS and cognitive performance. A star indicates significance at the 5% level after the Benjamini–Hochberg correction.

## 4. Discussion

We aimed to establish a depressive symptom severity threshold in BD that could be used to identify patients with depression‐associated cognitive impairment in clinical and research settings.

Consistent with prior studies, a DSM‐based diagnosis of depression was associated with poorer composite cognition. However, the effect of depression on global cognitive performance found in the present study was smaller than in the studies by Martínez‐Arán and collaborators, who reported a SMD between −0.5 and −0.8. This discrepancy is likely due to lower symptom severity in the outpatient sample recruited in the present study compared to the inpatient population they studied. Other studies have reported poorer executive function during bipolar depression [46]. The present findings diverge from prior studies suggesting that working memory [47] and processing speed performance [48] are not aggravated by bipolar depression. Contrary to expectations, no significant associations were observed between depression and attention [6, 8], possibly because attention impairments may occur in both depressed and nondepressed individuals with BD [10], masking group differences. While our results confirmed the validity of the DSM‐based diagnosis of depressive episode for identifying individuals with type I BD with depression‐induced cognitive impairment, these criteria may lack sensitivity and miss individuals with milder depressive symptoms affecting cognition. Individuals with type II BD more frequently exhibit preserved cognitive performance than those with type I BD [49]. Our findings additionally suggest that individuals with type II BD are either less susceptible to depression‐induced cognitive impairment or that such impairment appears at a higher threshold of depression severity than detected by DSM criteria.

A MADRS cutoff of ≥12 best classified DSM‐defined depression in our sample. This cut‐off is slightly lower than the threshold reported by the International Society for BDs, which recommends using a MADRS score > 14 to define syndromal depression [17]. This difference again reflects the lower depressive symptom severity in our sample compared to other samples of individuals with BD.

Interestingly, MADRS ≥ 12 also marked the level at which individuals had significantly lower composite cognitive performance compared to those with a MADRS score ≤ 1, suggesting that the DSM‐based diagnosis for a depressive episode matches the level of depression symptom severity at which composite cognition is significantly impaired. However, composite cognition in individuals scoring 12 or higher was similar to those scoring between 2 and 11, suggesting a continuous rather than threshold‐based relationship between depressive symptom severity and cognitive impairment. These results indicate that the significant cognitive differences between remitted and non‐remitted patients with BD are likely driven by individuals at the clinical extremes (i.e., euthymic versus highly depressed). For individuals with MADRS scores near the threshold of 12, cognitive performance is comparable, blurring the boundary between remission and depression. This is supported by the significant linear association we measured and prior cross‐sectional studies reporting linear associations between the MADRS score and worse cognitive performance in 1267 euthymic patients, with effect sizes ranging between −0.13 and −0.09 [13] and in 80 depressive patients with BD, with correlation coefficients ranging between −0.43 and −0.24 [50]. Therefore, we do not recommend using the total MADRS score in order to exclude individuals at high risk of depression‐induced cognitive impairment in BD.

Processing speed performance was significantly impaired in individuals with MADRS scores ≥ 5 versus those with scores ≤ 1. This result is in line with a previous cross‐sectional study reporting that shifting from none to mild depressive symptoms is associated with a 0.36‐increase in processing speed impairment in adults with BD [12]. Declines in processing speed may occur even with mild depressive symptoms. Our results suggest that processing speed is likely impaired at a lower depressive symptom severity than other cognitive domains. However, processing speed performance was statistically similar between individuals with MADRS scores ≥ 5 and those with scores between 2 and 4. Overall, we were unable to identify valid and relevant depression severity thresholds associated with significant cognitive impairment.

Specific symptoms may better explain cognitive impairments than overall depression severity. Concentration difficulty, apparent sadness, inability to feel, and lassitude were the most strongly associated with cognition, especially processing speed and executive function. Individuals with a positive sadness subscore were slightly cognitively impaired compared to other individuals, and this threshold only persisted in type I BD. Additionally, individuals with a detachment subscore (i.e., the sum of the scores for concentration difficulties, lassitude, and inability to feel) greater than 4 were slightly cognitively impaired compared with individuals with lower scores. This finding is in line with the associations between emotional withdrawal and blunted affect and worse global cognition, verbal learning, and executive function in mood and psychotic disorders [51]. Interestingly, this threshold seems to only apply to individuals with type II BD. Excluding individuals based on the detachment or sadness MADRS subscores would exclude 59.4% of individuals from our outpatient sample. Our results highlight that individuals with anhedonia, psychomotor retardation, or detachment, who carry higher risks for depression‐associated cognitive impairment, should be excluded from procognitive interventions and cognitive research targeting residual cognitive impairment in euthymic BD and could instead benefit from interventions targeting depression. Those findings additionally suggest that depression‐associated cognitive impairment has distinct patterns in type I and type II BD.

Unexpectedly, poor sleep was not associated with cognition, despite consistent reports of this link in previous studies [52, 53]. This item may be too specific to capture the broader relationship between sleep and cognition [52]. We found that worse executive function is significantly associated with suicidal ideation. This finding is consistent with research showing that impulsivity, which contributes to worse executive function, is significantly higher in individuals with BD or MDD with a history of suicide attempts than in those without [54].

Among covariates, having type II BD was associated with better composite cognition performance than type I, consistent with previous findings [6, 55]. A history of psychosis was associated with worse performance in composite cognition, though evidence on this association is mixed [56, 57].

This study had several limitations. First, we did not use the complete battery recommended by the International Society for BDs. Therefore, we missed the cognitive abilities measured by the Brief Visuospatial Memory Test–Revised or the Wisconsin Card Sorting Test, which might exhibit a threshold of depressive‐associated cognitive impairment. Conners’ Continuous Performance Test II V.5 was used instead of the Continuous Performance Test–Identical Pairs; thus, the measure of attention in the present study may be more related to cognitive impulsivity than to vigilance. Similarly, WAIS digit span was used instead of Wechsler Memory Scale–III Letter–Number Sequencing. Besides, we did not assess whether our data fitted the theoretical organization proposed by the battery. We could not differentiate between individuals with missing treatment data and those who never received any psychotropic medication. Cognitive performance is significantly worse in drug‐naive patients with BD before receiving a treatment compared to after [58], although those results might be confounded by indication. Other studies, however, suggest that untreated individuals exhibit cognitive performance similar to that of treated individuals [59, 60]. Additionally, we did not test any directional hypothesis between depressive symptoms and cognition, despite evidence of asymmetrical effects [61]. Longitudinal data would have enabled us to test such associations. Using a scale specifically targeting residual symptoms, such as the depression residual symptom scale [62], might have improved sensitivity for detecting depression‐associated cognitive impairment. Finally, our findings might be biased by unmeasured confounders such as lifestyle or familial factors.

Overall, impairments in composite cognitive performance, especially in processing speed, were consistently significantly associated with more severe depressive symptoms. We were unable to identify a relevant depression severity threshold for the total MADRS score, indicating depression‐induced cognitive impairment. Rather than participating in procognitive interventions or research intended for strictly euthymic individuals, patients with type I BD who have a sadness subscore > 0 or those with type II BD who have a detachment subscore ≥ 4 should first receive treatment for their depressive symptoms. Addressing these symptoms is a necessary precursor to focusing on residual cognitive impairments, as these individuals have likely depression‐induced cognitive decline. Besides, excluding individuals with a DSM‐based diagnosis of depression remains a convenient and valid method.

## Author Contributions

Paul Roux, Eric Brunet‐Gouet, Alexandre Billard, and Nathan Vidal defined the study’s objectives, designed the study, and conducted data processing, whereas data collection was overseen by the FACE‐BD collaborators including Solène Frileux, Raoul Belzeaux, Anamaria Bogdan, Philippe Courtet, Thierry D’Amato, Caroline Dubertret, Bruno Etain, Emmanuel Haffen, Dominique Januel, Marion Leboyer, Antoine Lefrere, Pierre‐Michel Llorca, Emeline Marlinge, Katia M’Bailara, Emilie Olié, Mircea Polosan, Raymund Schwan, and Michel Walter. Paul Roux, Nathan Vidal, Alexandre Billard, and Eric Brunet‐Gouet conducted the data analysis and wrote the manuscript. All authors participated in interpreting the results, editing the manuscript, and revising the figures.

## Funding

This study was supported by the Centre Hospitalier de Versailles (Bourse Registre 2019), the Fondation FondaMental, Créteil, France, and the Investissements d’Avenir Programs managed by the ANR (Grants ANR‐11‐IDEX‐0004‐02 and ANR‐10‐COHO‐10‐01).

## Disclosure

A preprint has previously been published [24].

## Ethics Statement

The local ethics committee (*Comité de Protection des Personnes Ile de France IX*) approved the study on January 18, 2010, under French law for noninterventional studies (observational studies without any risk, constraint, supplementary, or unusual procedure concerning diagnosis, treatment, or monitoring). The board required that all patients be given an informational letter but waived the requirement for written informed consent. However, verbal consent was witnessed and formally recorded.

## Conflicts of Interest

The authors declare no conflicts of interest.

## Supporting Information

Additional supporting information can be found online in the Supporting Information section.

## Supporting information


**Supporting Information** File S1: SM1: STROBE guidelines. Figure S1: Flowchart of the study. Figure S2: Performance of the MADRS total score to classify depressive episodes defined by the structured interview checking DSM criteria. The optimal cutoff value maximizing sensitivity and specificity is MADRS ≥ 11.5. Table S1: Full results of the association between major depression diagnosis based on the DSM criteria and the composite cognitive score. Analyses were run after multiple imputation. Table S2: Association between major depression diagnosis based on the DSM criteria and cognitive performance across BD subtype. Estimates were obtained in multiple linear regression models. Results for the covariates were not displayed for clarity. Table S3: Association between belonging to one of the MADRS score categories and composite cognitive performance in multiple linear regression models. Analyses were run with multiple imputation. Table S4: Effect associated with each contrast of the six categories of the MADRS total score in multiple linear regression models estimating cognitive performance (*n* = 2532). Covariates included education level, the history of psychotic symptoms, anticholinergic burden, the use of anxiolytics, anticonvulsants, antipsychotics, lithium, the use of any psychotropic treatment, time since the onset of the disorder, and a variable indicating whether the patient experienced more than one depressive episode. We corrected *p*‐values using the Benjamini–Hochberg method. Table S5: Effect associated with each contrast of the six categories of the MADRS total score in multiple linear regression models estimating cognitive performance across BD subtypes. Covariates included education level, the history of psychotic symptoms, anticholinergic burden, the use of anxiolytics, anticonvulsants, antipsychotics, lithium, the use of any psychotropic treatment, time since the onset of the disorder, and a variable indicating whether the patient experienced more than one depressive episode. We corrected *p*‐values using the Benjamini–Hochberg method. Table S6: Effect associated with each contrast of the six categories of the MADRS domain subscores in multiple linear regression models estimating composite cognitive performance (*n* = 2532). Covariates included education level, the history of psychotic symptoms, anticholinergic burden, the use of anxiolytics, anticonvulsants, antipsychotics, lithium, the use of any psychotropic treatment, time since the onset of the disorder, and a variable indicating whether the patient experienced more than one depressive episode. We corrected *p*‐values using the Benjamini–Hochberg method. Table S7: Effect associated with each contrast of the six categories of the MADRS domain subscores in multiple linear regression models estimating composite cognitive performance across BD subtypes. Covariates included education level, the history of psychotic symptoms, anticholinergic burden, the use of anxiolytics, anticonvulsants, antipsychotics, lithium, the use of any psychotropic treatment, time since the onset of the disorder, and a variable indicating whether the patient experienced more than one depressive episode. We corrected *p*‐values using the Benjamini–Hochberg method.

## Data Availability

Due to ethical and legal restrictions, data involving clinical participants cannot be made publicly available. All relevant data are available upon request to the Fondation FondaMental for researchers who meet the criteria for access to confidential data.
