# Supplementary material for: Is It Possible to Establish Thresholds for Depressive Symptom Severity Associated With Cognitive Impairments in Bipolar Disorders? Findings From the FACE‐BD Cohort
Source: Depress Anxiety. 2026 Oct 26;2026:9918396. doi: 10.1155/da/9918396 (PMC13698664; doi:10.1155/da/9918396)
Supplement: Supplementary file 1 [file DA-2026-9918396-s001.docx]

# Is it Possible to Establish Thresholds for Depressive Symptom Severity Associated with Cognitive Impairments in Bipolar Disorders ? Findings From the FACE-BD Cohort

Supplementary information

## Supplementary material SM1: STROBE guidelines.

|  | **Item No** | **Recommendation** | **Page No** |
| --- | --- | --- | --- |
| **Title and abstract** | 1 | (*a*) Indicate the study’s design with a commonly used term in the title or the abstract | 1 |
|  |  | (*b*) Provide in the abstract an informative and balanced summary of what was done and what was found | 4 |
| **Introduction** | | | |
| Background/rationale | 2 | Explain the scientific background and rationale for the investigation being reported | 5-6 |
| Objectives | 3 | State specific objectives, including any prespecified hypotheses | 6 |
| **Methods** | | | |
| Study design | 4 | Present key elements of study design early in the paper | 6 |
| Setting | 5 | Describe the setting, locations, and relevant dates, including periods of recruitment, exposure, follow-up, and data collection | 6 |
| Participants | 6 | (*a*) Give the eligibility criteria, and the sources and methods of case ascertainment and control selection. Give the rationale for the choice of cases and controls | 6 |
|  |  | (*b*) For matched studies, give matching criteria and the number of controls per case | NA |
| Variables | 7 | Clearly define all outcomes, exposures, predictors, potential confounders, and effect modifiers. Give diagnostic criteria, if applicable | 7-8 |
| Data sources/ measurement | 8* | For each variable of interest, give sources of data and details of methods of assessment (measurement). Describe comparability of assessment methods if there is more than one group | 7-8 |
| Bias | 9 | Describe any efforts to address potential sources of bias | 8-9 |
| Study size | 10 | Explain how the study size was arrived at | 9 |
| Quantitative variables | 11 | Explain how quantitative variables were handled in the analyses. If applicable, describe which groupings were chosen and why | 9 |
| Statistical methods | 12 | (*a*) Describe all statistical methods, including those used to control for confounding | 9 |
|  |  | (*b*) Describe any methods used to examine subgroups and interactions | 9 |
|  |  | (*c*) Explain how missing data were addressed | 9 |
|  |  | (*d*) If applicable, explain how matching of cases and controls was addressed | NA |
|  |  | (*e*) Describe any sensitivity analyses | 9 |
| **Results** | | | |
| Participants | 13* | (a) Report numbers of individuals at each stage of study—eg numbers potentially eligible, examined for eligibility, confirmed eligible, included in the study, completing follow-up, and analysed | Figure S1 |
|  |  | (b) Give reasons for non-participation at each stage | Figure S1 |
|  |  | (c) Consider use of a flow diagram | Figure S1 |
| Descriptive data | 14* | (a) Give characteristics of study participants (eg demographic, clinical, social) and information on exposures and potential confounders | 9 |
|  |  | (b) Indicate number of participants with missing data for each variable of interest | Table 1 & 2 |
| Outcome data | 15* | Report numbers in each exposure category, or summary measures of exposure | Table 1 & 2 |

| Main results | | 16 | (*a*) Give unadjusted estimates and, if applicable, confounder-adjusted estimates and their precision (eg, 95% confidence interval). Make clear which confounders were adjusted for and why they were included | Table 3, S2 et S3 |
| --- | --- | --- | --- | --- |
|  |  |  | (*b*) Report category boundaries when continuous variables were categorized | 9 |
|  |  |  | (*c*) If relevant, consider translating estimates of relative risk into absolute risk for a meaningful time period |  |
| Other analyses | 17 | Report other analyses done—eg analyses of subgroups and interactions, and sensitivity analyses | | Supplementary materials |
| **Discussion** | | | | |
| Key results | 18 | Summarise key results with reference to study objectives | | 12 |
| Limitations | 19 | Discuss limitations of the study, taking into account sources of potential bias or imprecision. Discuss both direction and magnitude of any potential bias | | 14 |
| Interpretation | 20 | Give a cautious overall interpretation of results considering objectives, limitations, multiplicity of analyses, results from similar studies, and other relevant evidence | | 12-14 |
| Generalisability | 21 | Discuss the generalisability (external validity) of the study results | | 14 |
| **Other information** | | | | |
| Funding | 22 | Give the source of funding and the role of the funders for the present study and, if applicable, for the original study on which the present article is based | | 15 |

*Give information separately for cases and controls.

**Note:** An Explanation and Elaboration article discusses each checklist item and gives methodological background and published examples of transparent reporting. The STROBE checklist is best used in conjunction with this article (freely available on the Web sites of PLoS Medicine at http://www.plosmedicine.org/, Annals of Internal Medicine at http://www.annals.org/, and Epidemiology at http://www.epidem.com/). Information on the STROBE Initiative is available at http://www.strobe-statement.org.

**Figure S1. Flowchart of the study.**

**Figure S2. Performance of the MADRS total score to classify depressive episodes defined by the structured interview checking DSM criteria.** The optimal cutoff value maximizing sensitivity and specificity is MADRS ≥11.5.

**Table S1. Full results of the association between major depression diagnosis based on the DSM criteria and the composite cognitive score.** Analyses were run after multiple imputation.

| **Category** | | ***β* (95% CI)** | **uncorrected *p*-value** | **fmi** |
| --- | --- | --- | --- | --- |
| Intercept | | **-0.79 (-0.60: -0.98)** | **<.001** | 0.014 |
| Major depression | no (ref) | - | - | - |
|  | yes | **-0.17 (-0.28: -0.05)** | **0.003** | 0.001 |
| Education level, years | | **0.05 (0.04: 0.06)** | **<.001** | 0.001 |
| BD subtype | BDI (ref) | - | - | - |
|  | BDII | **0.10 (0.02: 0.18)** | **<.001** | 0.060 |
|  | NOS | 0.03 (-0.07: 0.13) | 0.590 | 0.022 |
| History of psychosis | | -0.05 (-0.13: 0.03) | 0.249 | 0.180 |
| Anticholinergic burden | | -0.01 (-0.04: 0.02) | 0.652 | 0.001 |
| The use of anxiolytics | | **-0.09 (-0.16: -0.02)** | **0.015** | 0.002 |
| The use of lithium | | **-0.20 (-0.24: -0.16)** | **<.001** | 0.002 |
| The use of anticonvulsants | | -0.02 (-0.05: 0.01) | 0.469 | 0.002 |
| The use of antipsychotics | | **-0.27 (-0.34: -0.20)** | **<.001** | 0.003 |
| The use of antidepressants | | -0.004 (-0.012: 0.004) | 0.912 | 0.003 |
| The use of any treatment | | **0.14 (0.02: 0.26)** | **0.019** | 0.002 |
| Duration of the BD | 0-3 years (ref) | - | - | - |
|  | 4-10 years | -0.02 (-0.13: 0.09) | 0.789 | 0.083 |
|  | >10 years | 0.01 (-0.09: 0.11) | 0.788 | 0.052 |
| Experienced >1 depressive episode | | **0.14 (0.04: 0.28)** | **0.008** | 0.153 |

**Table S2. Association between major depression diagnosis based on the DSM criteria and cognitive performance across BD subtype.** Estimates were obtained in multiple linear regression models. Results for the covariates were not displayed for clarity.

| **Category** | ***n*** | | ***β* (95% CI)** | **Standardized *β*** | **adjusted *p-value*** | **fmi** |
| --- | --- | --- | --- | --- | --- | --- |
|  | **non-depressed** | **depressed** |  |  |  |  |
| **Type I Bipolar disorders** | | | | | | |
| Attention | 685 | 47 | -0.13 (-0.62: 0.36) | -0.09 | 0.609 | - |
| Executive function | 1125 | 87 | **-0.28 (-0.55: -0.01)** | **-0.26** | **0.043** | **0.004** |
| Processing speed | 1125 | 87 | **-0.17 (-0.34: 0.01)** | **-0.23** | **0.058** | **0.003** |
| Verbal memory | 1125 | 87 | -0.24 (-0.55: 0.07) | -0.18 | 0.127 | 0.018 |
| Working memory | 1125 | 87 | **-0.29 (-0.51: -0.06)** | **-0.31** | **0.012** | **0.007** |
| Composite cognition | 1125 | 87 | **-0.23 (-0.4: -0.06)** | **-0.31** | **0.009** | **0.003** |
| Verbal comprehension and perceptual reasoning | 1125 | 87 | 0.07 (-0.19: 0.33) | 0.07 | 0.6 | 0.226 |
| **Type II Bipolar disorders** | | | | | | |
| Attention | 549 | 47 | 0.17 (-0.4: 0.74) | 0.12 | 0.567 | - |
| Executive function | 952 | 102 | -0.18 (-0.42: 0.05) | -0.17 | 0.13 | 0.019 |
| Processing speed | 952 | 102 | -0.07 (-0.23: 0.1) | -0.09 | 0.423 | 0.02 |
| Verbal memory | 952 | 102 | 0.1 (-0.2: 0.41) | 0.08 | 0.511 | 0.035 |
| Working memory | 952 | 102 | -0.16 (-0.36: 0.05) | -0.17 | 0.134 | 0.02 |
| Composite cognition | 952 | 102 | -0.07 (-0.23: 0.08) | -0.1 | 0.367 | 0.018 |
| Verbal comprehension and perceptual reasoning | 952 | 102 | 0.09 (-0.16: 0.34) | 0.09 | 0.481 | 0.204 |

**Table S3. Association between belonging to one of the MADRS score categories and composite cognitive performance in multiple linear regression models.** Analyses were run with multiple imputation.

| **Category** | | ***β* (95% CI)** | **uncorrected *p*-value** | **fmi** |
| --- | --- | --- | --- | --- |
| Intercept | | **-0.79 (-1.01: -0.57)** | **<.001** | 0.018 |
| MADRS score | ≤ 1 (ref) | - | - | - |
|  | 2-4 | -0.04 (-0.14: 0.06) | 0.395 | 0.002 |
|  | 5-7 | -0.10 (-0.20: 0) | 0.058 | 0.002 |
|  | 8-11 | -0.10 (-0.20: 0) | 0.071 | 0.003 |
|  | 12-17 | **-0.16 (-0.26: -0.06)** | **0.003** | 0.005 |
|  | ≥ 18 | **-0.15 (-0.25: -0.05)** | **0.005** | 0.003 |
| Education level, years | | **0.05 (0.04: 0.06)** | **<.001** | 0.003 |
| BD subtype | BDI (ref) | **-** | **-** | - |
|  | BDII | **0.10 (0.02: 0.18)** | **0.002** | 0.066 |
|  | NOS | 0.04 (-0.14: 0.22) | 0.541 | 0.022 |
| History of psychosis | | -0.06 (-0.14: 0.02) | 0.190 | 0.189 |
| Anticholinergic burden | | -0.03 (-0.09: 0.03) | 0.246 | 0.002 |
| The use of anxiolytics | | **-0.09 (-0.15: -0.03)** | **0.016** | 0.002 |
| The use of lithium | | **-0.23 (-0.31: -0.15)** | **<.001** | 0.002 |
| The use of anticonvulsants | | -0.06 (-0.14: 0.02) | 0.097 | 0.002 |
| The use of antipsychotics | | **-0.29 (-0.37: -0.21)** | **<.001** | 0.003 |
| The use of antidepressants | | 0 (-0.04: 0.04) | 0.927 | 0.004 |
| The use of any treatment | | **0.14 (0.02: 0.26)** | **0.026** | 0.002 |
| Duration of the BD | 0-3 years (ref) | **-** | **-** | - |
|  | 4-10 years | -0.01 (-0.13: 0.11) | 0.872 | 0.083 |
|  | >10 years | 0.02 (-0.08: 0.12) | 0.661 | 0.053 |
| Experienced >1 depressive episode | | **0.15 (0.05: 0.25)** | **0.005** | 0.151 |

**Table S4. Effect associated with each contrast of the six categories of the MADRS total score in multiple linear regression models estimating cognitive performance (*n* = 2,532).** Covariates included education level, the history of psychotic symptoms, anticholinergic burden, the use of anxiolytics, anticonvulsants, antipsychotics, lithium, the use of any psychotropic treatment, time since the onset of the disorder, and a variable indicating whether the patient experienced more than one depressive episode. We corrected *p*-values using the Benjamini-Hochberg method.

| **Composite cognition** | **MADRS ≤ 1** | **2 ≤ MADRS ≤ 4** | **5 ≤ MADRS ≤ 7** | **8 ≤ MADRS ≤ 11** | **12 ≤ MADRS ≤ 17** | **MADRS ≥ 18** |
| --- | --- | --- | --- | --- | --- | --- |
| **MADRS ≤ 1** | - | -0.04 | -0.10 | -0.10 | **-0.16*** | **-0.15*** |
| **2 ≤ MADRS ≤ 4** | - | - | -0.06 | -0.05 | -0.12 | -0.11 |
| **5 ≤ MADRS ≤ 7** | - | - | - | 0.01 | -0.06 | -0.05 |
| **8 ≤ MADRS ≤ 11** | - | - | - | - | -0.06 | -0.05 |
| **12 ≤ MADRS ≤ 17** | - | - | - | - | - | 0.01 |
| **MADRS ≥18** | - | - | - | - | - | - |
| **Executive function Processing speed** | **MADRS ≤ 1** | **2 ≤ MADRS ≤ 4** | **5 ≤ MADRS ≤ 7** | **8 ≤ MADRS ≤ 11** | **12 ≤ MADRS ≤ 17** | **MADRS ≥ 18** |
| **MADRS ≤ 1** | - | -0.03 | -0.03 | -0.12 | -0.20 | -0.21 |
| **2 ≤ MADRS ≤ 4** | -0.12 | - | -0.01 | -0.10 | -0.17 | -0.18 |
| **5 ≤ MADRS ≤ 7** | -0.13 | -0.02 | - | -0.09 | -0.16 | -0.17 |
| **8 ≤ MADRS ≤ 11** | **-0.15*** | -0.03 | -0.02 | - | -0.07 | -0.08 |
| **12 ≤ MADRS ≤ 17** | **-0.21**** | -0.09 | -0.08 | -0.06 | - | -0.01 |
| **MADRS ≥18** | **-0.16*** | -0.05 | -0.03 | -0.01 | 0.05 | - |
| **Verbal memory Attention** | **MADRS ≤ 1** | **2 ≤ MADRS ≤ 4** | **5 ≤ MADRS ≤ 7** | **8 ≤ MADRS ≤ 11** | **12 ≤ MADRS ≤ 17** | **MADRS ≥ 18** |
| **MADRS ≤ 1** | - | 0.04 | -0.13 | -0.11 | -0.25 | -0.13 |
| **2 ≤ MADRS ≤ 4** | -0.08 | - | -0.17 | -0.15 | **-0.29*** | -0.16 |
| **5 ≤ MADRS ≤ 7** | -0.18 | -0.10 | - | 0.02 | -0.12 | 0.01 |
| **8 ≤ MADRS ≤ 11** | -0.15 | -0.07 | 0.02 | - | -0.14 | -0.01 |
| **12 ≤ MADRS ≤ 17** | -0.21 | -0.12 | -0.02 | -0.05 | - | 0.13 |
| **MADRS ≥18** | -0.25 | -0.17 | -0.07 | -0.09 | -0.04 | - |
| **Working memory Verbal comprehension and perceptual reasoning** | **MADRS ≤ 1** | **2 ≤ MADRS ≤ 4** | **5 ≤ MADRS ≤ 7** | **8 ≤ MADRS ≤ 11** | **12 ≤ MADRS ≤ 17** | **MADRS ≥ 18** |
| **MADRS ≤ 1** | - | -0.05 | -0.10 | -0.01 | -0.01 | -0.09 |
| **2 ≤ MADRS ≤ 4** | -0.14 | - | -0.05 | 0.04 | 0.04 | -0.04 |
| **5 ≤ MADRS ≤ 7** | -0.01 | 0.12 | - | 0.10 | 0.10 | 0.01 |
| **8 ≤ MADRS ≤ 11** | 0.03 | 0.16 | 0.04 | - | 0 | -0.08 |
| **12 ≤ MADRS ≤ 17** | -0.01 | 0.13 | 0.01 | -0.03 | - | -0.08 |
| **MADRS ≥18** | 0.08 | 0.21 | 0.09 | 0.05 | 0.09 | - |

**0.01 < p < 0.05; **0.001 < p ≤ 0.01; ***p < 0.001*

**Table S5. Effect associated with each contrast of the six categories of the MADRS total score in multiple linear regression models estimating cognitive performance across BD subtypes.** Covariates included education level, the history of psychotic symptoms, anticholinergic burden, the use of anxiolytics, anticonvulsants, antipsychotics, lithium, the use of any psychotropic treatment, time since the onset of the disorder, and a variable indicating whether the patient experienced more than one depressive episode. We corrected *p*-values using the Benjamini-Hochberg method.

| **Type I BD** | | | | | | |
| --- | --- | --- | --- | --- | --- | --- |
| **Composite cognition**  **Processing speed** | **MADRS ≤ 1** | **2 ≤ MADRS ≤ 4** | **5 ≤ MADRS ≤ 7** | **8 ≤ MADRS ≤ 11** | **12 ≤ MADRS ≤ 17** | **MADRS ≥ 18** |
| **MADRS ≤ 1** | - | -0.07 | -0.10 | -0.12 | -0.21 | -0.09 |
| **2 ≤ MADRS ≤ 4** | -0.07 | - | -0.03 | -0.05 | -0.15 | -0.02 |
| **5 ≤ MADRS ≤ 7** | -0.10 | -0.03 | - | -0.03 | -0.12 | 0.01 |
| **8 ≤ MADRS ≤ 11** | -0.12 | -0.05 | -0.03 | - | -0.09 | 0.03 |
| **12 ≤ MADRS ≤ 17** | -0.21 | -0.14 | -0.11 | -0.09 | - | 0.12 |
| **MADRS ≥18** | -0.09 | -0.02 | 0.01 | 0.03 | 0.12 | - |
| **Type II BD** | | | | | | |
| **Composite cognition**  **Processing speed** | **MADRS ≤ 1** | **2 ≤ MADRS ≤ 4** | **5 ≤ MADRS ≤ 7** | **8 ≤ MADRS ≤ 11** | **12 ≤ MADRS ≤ 17** | **MADRS ≥ 18** |
| **MADRS ≤ 1** | - | 0.02 | -0.01 | -0.06 | -0.05 | -0.13 |
| **2 ≤ MADRS ≤ 4** | -0.14 | - | -0.03 | -0.08 | -0.07 | -0.15 |
| **5 ≤ MADRS ≤ 7** | -0.09 | 0.05 | - | -0.05 | -0.04 | -0.12 |
| **8 ≤ MADRS ≤ 11** | -0.11 | 0.03 | -0.01 | - | 0.01 | -0.07 |
| **12 ≤ MADRS ≤ 17** | -0.17 | -0.03 | -0.08 | -0.07 | - | -0.08 |
| **MADRS ≥18** | -0.15 | -0.01 | -0.06 | -0.04 | 0.03 | - |

**Table S6. Effect associated with each contrast of the six categories of the MADRS domain subscores in multiple linear regression models estimating composite cognitive performance (*n* = 2,532).** Covariates included education level, the history of psychotic symptoms, anticholinergic burden, the use of anxiolytics, anticonvulsants, antipsychotics, lithium, the use of any psychotropic treatment, time since the onset of the disorder, and a variable indicating whether the patient experienced more than one depressive episode. We corrected *p*-values using the Benjamini-Hochberg method.

| Sadness subscore^1^ | 0  (*n* = 1,280) | 1 to 3  (*n* = 680) | 4 to 12  (*n* = 572) |
| --- | --- | --- | --- |
| 0 | - | **-0.10*** | **-0.12**** |
| 1 to 3 | - | - | -0.02 |
| Neurovegetative subscore^2^ | 0  (*n* = 798) | 1 to 3  (*n* = 955) | 4 to 12  (*n* = 779) |
| 0 | - | -0.04 | -0.03 |
| 1 to 3 | - | - | 0.01 |
| Detachment subscore^3^ | 0  (*n* = 723) | 1 to 3  (*n* = 752) | 4 to 12  (*n* = 1057) |
| 0 | - | -0.07 | **-0.16***** |
| 1 to 3 | - | - | **-0.09*** |
| Negative thoughts subscore^4^ | 0  (*n* = 1,287) | 1 to 3  (*n* = 640) | 4 to 12  (*n* = 605) |
| 0 | - | -0.03 | -0.06 |
| 1 to 3 | - | - | -0.03 |

*^1^Sum of the scores of apparent sadness and reported sadness.*

*^2^Sum of the scores of inner tension, reduced sleep, and reduced appetite.*

*^3^Sum of the scores of concentration difficulties, lassitude, and inability to feel.*

*^4^Sum of the scores of pessimistic thoughts and suicidal thoughts.*

**0.01 < p < 0.05; **0.001 < p ≤ 0.01; ***p < 0.001*

**Table S7. Effect associated with each contrast of the six categories of the MADRS domain subscores in multiple linear regression models estimating composite cognitive performance across BD subtypes.** Covariates included education level, the history of psychotic symptoms, anticholinergic burden, the use of anxiolytics, anticonvulsants, antipsychotics, lithium, the use of any psychotropic treatment, time since the onset of the disorder, and a variable indicating whether the patient experienced more than one depressive episode. We corrected *p*-values using the Benjamini-Hochberg method.

| Type I BD | | | |
| --- | --- | --- | --- |
| Sadness subscore^1^ | 0  (*n* = 682) | 1 to 3  (*n* = 316) | 4 to 12  (*n* = 214) |
| 0 | - | **-0.16**** | -0.13 |
| 1 to 3 | - | - | 0.03 |
| Neurovegetative subscore^2^ | 0  (*n* = 447) | 1 to 3  (*n* = 483) | 4 to 12  (*n* = 282) |
| 0 | - | -0.04 | -0.05 |
| 1 to 3 | - | - | -0.01 |
| Detachment subscore^3^ | 0  (*n* = 378) | 1 to 3  (*n* = 382) | 4 to 12  (*n* = 452) |
| 0 | - | -0.10 | -0.12 |
| 1 to 3 | - | - | -0.02 |
| Negative thoughts subscore^4^ | 0  (*n* = 679) | 1 to 3  (*n* = 393) | 4 to 12  (*n* = 140) |
| 0 | - | -0.04 | 0 |
| 1 to 3 | - | - | 0.04 |
| Type II BD | | | |
| Sadness subscore^1^ | 0  (*n* = 478) | 1 to 3  (*n* = 292) | 4 to 12  (*n* = 284) |
| 0 | - | -0.03* | -0.08 |
| 1 to 3 | - | - | -0.05 |
| Neurovegetative subscore^2^ | 0  (*n* = 276) | 1 to 3  (*n* = 378) | 4 to 12  (*n* = 400) |
| 0 | - | 0.04 | 0.02 |
| 1 to 3 | - | - | -0.02 |
| Detachment subscore^3^ | 0  (*n* = 276) | 1 to 3  (*n* = 287) | 4 to 12  (*n* = 491) |
| 0 | - | 0 | **-0.17**** |
| 1 to 3 | - | - | **-0.17**** |
| Negative thoughts subscore^4^ | 0  (*n* = 489) | 1 to 3  (*n* = 357) | 4 to 12  (*n* = 208) |
| 0 | - | -0.05 | -0.09 |
| 1 to 3 | - | - | -0.04 |

*^1^Sum of the scores of apparent sadness and reported sadness.*

*^2^Sum of the scores of inner tension, reduced sleep, and reduced appetite.*

*^3^Sum of the scores of concentration difficulties, lassitude, and inability to feel.*

*^4^Sum of the scores of pessimistic thoughts and suicidal thoughts.*

**0.01 < p < 0.05; **0.001 < p ≤ 0.01; ***p < 0.001*
